# Supplementary material for: Nasal vs. oral BREATHing WIn Strategies in healthy individuals during cardiorespiratory Exercise testing (BreathWISE)
Source: PLoS One. 2025 Jul 16;20(7):e0326661. doi: 10.1371/journal.pone.0326661 (PMC12266398; doi:10.1371/journal.pone.0326661)
Supplement: Attachment — Submitted filename: PONE-D-24-54440 rev JR.pdf [file pone.0326661.s002.pdf]

The article entitled **"Nasal vs. Oral BREATHing Win Strategies in Healthy Individuals during Cardiorespiratory Exercise Testing (BREATHWISE)",** is concerned with the alterations in ventilation and metabolism that occur in response to the partial or complete obstruction either of the nasal or oral airways during an exercise test. The main point of this article is the intervention on the nose and the mouth of humans to change the ventilation pattern during incremental exercise tests. This intervention aims to alter normal breathing during exercise.
The interest of this paper is to deal with the time to exchange respiratory gases, induced by higher flow resistance through the nasal pathway, and its possible application to air pathway features in pathology such as COPD or yoga or tai-chi strategies. Due to the characteristics of the CPETs carried out and their results data shown scarcely apply to athletic performance

***Major concerns***

They recruited six men and six women, a small sample size which makes it difficult to extrapolate the data to the general population. The data from the exercise tests show that the subjects were not in good physical condition (VO2 and VE), what left real maximal intensities unexplored. Furthermore, as occlusion of either the nose or mouth changes airflow resistance, a measurement of this variable at rest and during exercise is missing. As the nasal pathway represents two-thirds of the total resistance of the upper airways, occlusion of the mouth obviously increases upper airway resistances and consequently changes the ventilation pattern. Again there is a lack of recording of airflow characteristics such as ventilatory peak flow (i.e.TV/ti, TV/te), average flow, and tidal volume time constant. The authors do not report the predetermination of the sample size. From a statistical view, authors show mean ± standard deviation data but they do not explain if mean and SD correspond to each subject during every condition or, contrariwise, the whole group of experimental subjects. Since they examined 6 men and 6 women it is important to analyze women separately from men otherwise the smaller ventilatory volumes and the higher ventilatory frequency of women can introduce a bias in the results. Statistical data also must include the confidence interval for every variable and in every category. The above weakens the structure of this paper
.

***Minor concerns***

**Title**

#1-In the title appear Win and BreathWISE, both terms are not explained in the rest of the article. At least, Breath WISE should be explained as a structured approach to breathing techniques aimed at improving lung function, reducing stress and enhancing overall well-being. Contrariwise, both terms should be changed for “breathing”.

#2- It is not well described whether the specific strategies lead to an improvement in exercise performance or whether they have been directed towards breathing therapies.

#3- In the Abstract, the sentences “Twelve healthy subjects (28.6±5.2 y, 50% males) performed the 4 CPETs within one month. Variables were analysed at rest, at anaerobic threshold (AT), at intermediate exercise steps, and at peak” that appear in **Results,** should be move to **Methods in Abstract.**

#4- In the fifth line of the **Results** paragraph, in the Abstract, authors write “Moreover, peak inspiration and peak expiration time were augmented, while forced expiratory volume and vital capacity at rest were reduced. On opposite, only minor differences were detected at rest o AT”. If the variables forced expiratory volume and vital capacity were reduced at rest, what is the meaning of “On opposite, only minor differences were detected at rest or AT”? The authors should consider

explaining or deleting this sentence. Also the terms peak inspiration and peak expiratory time might be changed for "inspiratory and expiratory time to peak" for the sake of clarity.

#5- In **Methods**, the experimental subjects performed exercise in a cycle ergometer in which the subjects were allowed to see "rpm" (revolution per minute) but author did not tell if the rpm value was the same for the group or was different for each subjects or was fixed during the four conditions. It ought be clarified.

In *statistical analysis* the authors write that when the distribution of data belonged to a normal population they used mean±SD and when data belonged to a non-normal distribution they used median and interquartile. However normal and non-normal distributions were not assigned to the variables analysed in the article. It seems a stock phrase for statistic more than actual analysis.

#6- **RESULTS**: The values of VO2 peak and VCO2 peak reveal a low training status of the subjects. Attending to VO2 and VCO2 peaks the tests should be classified as submaximal. Also HR peaks did not reach the maximum corresponding to the average age of the sample. The workload peak around 200 W is not a value of athletes. By other side, it is known that women show higher HR than men what may introduce a bias in the statiscal analysis of this variable.

#7 **Figure 2**. Both panels are lacking the units in the ordinate axis. In FEV1 panel the ordinate values seem to be in percentage and FVC in litres; a Tiffenau index would be welcome. In the lower panel the ordinate scales should be in the same range to allow values to be compared. Provided the values of iT and eT (units?) in STD, pNAS and eOR conditions is difficult to believe in the significant differences among them, given the small sample and the standard deviation magnitudes. Further, in addition to the longer time for expiration as compared with inspiration, does not seem to there be significant differences in the eT values, including the eT peak value.

Provided the ventilatory frequency (30.43 in eNAS vs 40 in eOR) and the PetCO2 peak (45 in eNAS vs 36.92 in eOR) of the eNAS condition (Table 2), it is not easy to explain how an increase in the end tidal CO2 is not correlated with higher ventilatory frequency in eNAS. It is well known than increase CO2 undergo an increase in ventilatory frequency. An eT longer than iT can help to explain the higher PetCO2 value in eNAS condition but not the change in ventilator frequency. May be these data deserve a better. explanation.

#8- **Figure 4** The statistical differences applied to each workload step have no sense because increasing workload between steps (like Bruce Protocol) is mandatory. Moreover, this does not seem the main objective of the article but the different ventilation pattern among categories.. However, does there not be differences between each condition during the three first workload steps (in X axis). At least, differences between the eNAS and the other conditions in the last step of the CPET are clear. Again, the PetCO2 and the VE values deserve a better explanation.
