# Supplementary material for: Nasal vs. oral BREATHing WIn Strategies in healthy individuals during cardiorespiratory Exercise testing (BreathWISE)
Source: PLoS One. 2025 Jul 16;20(7):e0326661. doi: 10.1371/journal.pone.0326661 (PMC12266398; doi:10.1371/journal.pone.0326661)
Supplement: Attachment — Submitted filename: Reply to Rev2.docx [file pone.0326661.s001.docx]

**Response to the Reviewers**

**Manuscript: Nasal vs. oral BREATHing WIn Strategies in healthy individuals during cardiorespiratory Exercise testing (BreathWISE)**

**PLOS ONE (PONE-D-24-54440)**

Editor’s comment

Thank you for choosing PLOS ONE for the submission of your manuscript.

Your manuscript has been carefully assessed by three experienced reviewers who dedicated their time and effort to provide thorough, constructive, and insightful feedback.

We are grateful for their expertise in ensuring a rigorous review process.

Following this review, they have identified several concerns (MAJOR REVISION) that impact the overall strength and clarity of the study

**We thank the Editor for the possibility to revise our manuscript and for the positive comments received.**

**We acknowledge the effort of the 3 Reviewers and are very grateful for their positive and constructive comments. We have prepared a step by step reply and a new version of the paper in accordance to the comments received. We hope that they are satisfactorily and we believe that the manuscript is now improved.**

Reviewer #1: The paper by Mapelli and colleagues addresses a curious and interesting topic in exercise and functional capacity.
The paper is well written, but I would make some minor changes.
**We thank the Reviewer#1 for his positive feedback and for the suggestions provided. The manuscript has been modified accordingly (see below the point-by-point answers).**

- A recent paper by Eser P et al published in Front Physiol 2024 Aug 6;15:1380562 addresses a similar topic: I would discuss differences and added value of your paper compared with data already in the literature.

**Thank you for this important comment. We were not aware of this manuscript when we submitted the manuscript but we read it few weeks later. It is also very interesting and address similar topics. However, we believe that the 2 cohort and the methods differ significantly. In particular, we enrolled only healthy subjects and not patients and – more importantly – they exercised patient at a submaximal level (50% of the peak) while we performed maximal CPET. Indeed, comparing our data at 50% of the exercise (see Figure 4), we also did not find significant differences in term of VO2 and VE, as expressed in the “Young control subjects” by Eser et al. (see their Table 3, last columns, in which they also found a very minimal difference – 0.5 L/min – in VE). According to your comment we have expanded the discussion also adding the citation. Specifically, we now report “*These findings are in line with previous data recently published by Eser et al. 24 in a cohort of both healthy and heart failure patients. In their experiment, exercising the subjects at 50% of the maximal exercise in a 5-minutes constant workload testing, they observed very negligible differences in VE with eOR vs eNAS conditions (-0.5 L/min in the healthy subject’s group). These data, even in presence of slightly different experimental conditions (maximal incremental CPET vs submaximal workload test) suggests once again, the presence of more pronounced differences at higher exercise stages, as illustrated in the Figure 4 curves. This may suggest even greater differences in the presence of higher exercise intensities, as could occur in highly trained athletes, although this has not been investigated in the present study*.”**

-Some published work shows the importance of nasal breathing in terms of functional capacity (Tong TK, Effect of nostril dilatation on prolonged all-out intermittent exercise performance. J Sports Med Phys Fitness. 2001; Rappelt L, Front Physiol. 2023 Apr 20;14:1134778): could the fact that you found no differences in exclusively nasal breathing be because the nasal cycle was not considered? I would include among the limitations of the study this consideration.

**Thank you for this comment. We have added a dedicated sentence to the limitation section as suggested: *“Although not directly aimed to compare different voluntary breathing techniques, our study suggests that the wisest breathing strategy during an incremental exercise would involve a gradual increase in the oral contribution to VE from the AT until the end of the effort. These preliminary data may act as starting point for further studies exploring the role of different protocols of exercise training involving different breathing techniques aimed to improved exercise performance.”***

Reviewer #2: Many thanks for the opportunity to peer review this article. Reviewing the literature, I agree with the author this work adds support to the findings of the other literature highlighted in the discussion. The number of participants in this study is low however this limitation is acknowledged by the author and twelve subjects could be felt to be comparable to the numbers recruited in the other studies referred to in the text i.e. Morton et al. (1995) with 20 subjects, LaComb et al. (2017) with 19 subjects. In brief the study found that a breathing technique which involved just nasal respiration referred to as the eNAS condition caused a significant impairment in exercise performance during cardiopulmonary exercise testing (CPET).

**We really thank the Reviewer#2 for his nice feedback and for his comments. The manuscript has been modified accordingly (see below the point-by-point answers). We believe that following your suggestions it significantly improved.**

My recommendations are as follows:

Major: The authors do not make available all their data a requirement of the data availability section of the PLOS One submission. I quote: “Authors are required to make all data underlying the findings described fully available, without restriction, and from the time of publication. PLOS allows rare exceptions to address legal and ethical concerns” The authors response to this question is No- some restrictions will apply with Repository of raw data available after acceptance: [www.zenodo.org](https://urlsand.esvalabs.com/?u=http%3A%2F%2Fwww.zenodo.org&e=8c00e339&h=0ca16955&f=y&p=y) upon request to [direzione.scientifica@cardiologicomonzino.it](mailto:direzione.scientifica@cardiologicomonzino.it).

**We will change the access to the repository as “without any restriction” as requested.**

Minor: The article does contain some typos and inconsistent language for example the subject is referred to as subjects, participants and in other situations as patients. The term patient particularly should be avoided in the text and the article could benefit from more specifics on the inclusion/exclusion criteria.

**You are right and we are sorry. Indeed, the term “patients” was present mostly in the methods section since usually we performed CPET in HF patients. We have fixed the text leaving only the terms “subjects” or “participants” that are more appropriate**. **Thank you**

An aspect I found particularly vague in the methodology is the statement the ‘absence of lateral air leakage was carefully verified as a standard used procedure in CPET laboratories before each test.’ What is this standard used procedure in CPET laboratories? please either reference or describe the methodology.

**Thank you. According to your comment, we have modified the text and added a specific reference: *“The absence of lateral air leakage was carefully verified as a standard used procedure in CPET laboratories before each test by, completely blocking the ventilation valve of the spirometry mask with the palm of the hand as previously described ^11^.”***

Additionally, the statement all participants signed a written informed consent, and the protocol was approved by the local Ethics Committee (R1925/24 – L2-129) in my opinion should come at the beginning of the methods as signing informed consent form was on their inclusion criteria so it is slightly repetitive.

**Done as requested. Thank you**

Furthermore, the author states CPET data were analyzed breath by breath except peak VO2 analysis (averaged 20 seconds) yet earlier the author states: peak VO2 was calculated as the 30 seconds average of the highest VO2 recorded.

**You are right, the right value is 30 seconds. 20 was written by mistake. We fixed it in the text. Thank you.**

The statement: a maximal or nearly maximal effort was reached, as confirmed by RER>1.05 in all cases. Please reference the ERS statement for criteria of using RER>1.05 for a maximal test.

**Thank you. We have added it in the methods section**

It would also be beneficial to add heart rate data to further support these tests being maximal with a maximal test often being a peak heart rate >100% in adults which can be referenced from the same statement.
(Radtke et al., 2019) RADTKE, T., CROOK, S., KALTSAKAS, G., LOUVARIS, Z., BERTON, D., URQUHART, D. S., KAMPOURAS, A., RABINOVICH, R. A., VERGES, S., KONTOPIDIS, D., BOYD, J., TONIA, T., LANGER, D., DE BRANDT, J., GOËRTZ, Y. M. J., BURTIN, C., SPRUIT, M. A., BRAEKEN, D. C. W., DACHA, S., FRANSSEN, F. M. E., LAVENEZIANA, P., EBER, E., TROOSTERS, T., NEDER, J. A., PUHAN, M. A., CASABURI, R., VOGIATZIS, I. & HEBESTREIT, H. 2019. ERS statement on standardisation of cardiopulmonary exercise testing in chronic lung diseases. Eur Respir Rev, 28.
**As suggested, we have added the percent of predicted value of HR in table 2, and modified the method section accordingly: “*A maximal RER during exercise exceeding 1.05 was considered an indicator of a maximal effort ^17^. Similarly, a maximal peak HR above 100% was also used as a supportive criterion ^17-19^.”* As you can see, all the subject reached at least the 85% of their predicted HR (a more conservative cut-off used to indicate maximal exercise in the literature), but with average values far more than 95%. We have modified the text accordingly.**

Finally, within the discussion there is the statement within the limitations section suggesting data may not be reproducible in subjects trained to exercise with nose (or Mouth) occlusion in the prior weeks as evidenced in previous studies. Please reference/refer to the studies to support this statement.

**Thank you, added as requested, also according to the other Reviewer’s comments. We have also re-phrased that part of the text for clarity. Specifically*:” Therefore, these data may not be reproducible in subjects trained to exercise with nose (or mouth) occlusion in the prior weeks, neither with nasal dilators as evidenced in previous studies^27^. Moreover, as suggested by Tong et. al, a reduction in nasal resistance could affect exercise performance ^27-29^ but this aspect was not considered in the current trial. Similarly, elite athletes may exhibit different behaviours under the same circumstances; future trials conducted on this population may be helpful.”***

Reviewer #3: The article entitled "Nasal vs. Oral BREATHing Win Strategies in Healthy Individuals during Cardiorespiratory Exercise Testing (BREATHWISE)", is concerned with the alterations in ventilation and metabolism that occur in response to the partial or complete obstruction either of the nasal or oral airways during an exercise test. The main point of this article is the intervention on the nose and the mouth of humans to change the ventilation pattern during incremental exercise tests. This intervention aims to alter normal breathing during exercise.
The interest of this paper is to deal with the time to exchange respiratory gases, induced by higher flow resistance through the nasal pathway, and its possible application to air pathway features in pathology such as COPD or yoga or tai-chi strategies. Due to the characteristics of the CPETs carried out and their results data shown scarcely apply to athletic performance

**Thank you for your positive feedback and your insightful comments. We agree that our results are not entirely applicable to athletic subjects, since we enrolled an unselected population of healthy individuals. Further studies on athletes would be desirable, and this is now added to the limitation section.**

Major concerns
They recruited six men and six women, a small sample size which makes it difficult to extrapolate the data to the general population. The data from the exercise tests show that the subjects were not in good physical condition (VO2 and VE), what left real maximal intensities unexplored.

**Thank you for your comment. We agree that the physical condition of our individuals is not perfect, but definitely within a normal range (average VO_2_ in the STD condition is 33.4 ml/kg/min, corresponding to 90% of the predicted). However, as mentioned before, our aim was not to describe the effects of different respiration strategies in athletes, but only in the general healthy population. Following your suggestions, the text has been modified in the introduction as well as in the discussion and limitations sections. Specifically: “This may suggest even greater differences in the presence of higher exercise intensities, as could occur in highly trained athletes, although this has not been investigated in the present study.” ….and …”elite athletes may exhibit different behaviors under the same circumstances; future trials conducted on this population may be helpful”**

Furthermore, as occlusion of either the nose or mouth changes airflow resistance, a measurement of this variable at rest and during exercise is missing. As the nasal pathway represents two-thirds of the total resistance of the upper airways, occlusion of the mouth obviously increases upper airway resistances and consequently changes the ventilation pattern. Again, there is a lack of recording of airflow characteristics such as ventilatory peak flow (i.e.TV/ti, TV/te), average flow, and tidal volume time constant.

**I totally agree with your comment that eNAS have a higher airflow resistance and this explains the reduced ventilation observed in this condition (please see reply to your last comment below) and the more prolonged Ti. It is also true that we did not report some ventilation measurements such as TV/Ti and TV/te. However, TV and inspiratory and expiratory time were reported independently (see figure 2). I think that, to avoid overwhelming the reader with too many measurements there is no need to add these values. Please, note that the exercise protocol was a ramp (progressively increasing workload) so that it is not possible to calculate the time constant.**

The authors do not report the predetermination of the sample size.

**Sample size evaluation has been added to the statistical paragraph as you requested. Specifically: “Sample size determination: With a sample of 12 subjects we planned to identify an effect size of 1.2, with a power of 80%, considering an alpha=0.05 and an ANOVA for repeated measures (4 measurements).”**

From a statistical view, authors show mean ± standard deviation data but they do not explain if mean and SD correspond to each subject during every condition or, contrariwise, the whole group of experimental subjects. Since they examined 6 men and 6 women it is important to analyze women separately from men otherwise the smaller ventilatory volumes and the higher ventilatory frequency of women can introduce a bias in the results. Statistical data also must include the confidence interval for every variable and in every category. The above weakens the structure of this paper.

**Data are expressed as mean and SD as all tested variables in our study were normally distributed. Reported means refers to all patients in each condition. I agree that women and men have different ventilatory volumes, but as all the analysis were paired (within subject) we believe that results obtained are still meaningful. However, you are completely right and further studies with greater number of subjects would be needed to appreciate sex related differences.**
**As we completely agree with your comment we already included this aspect as a limitation in the text.**

Minor concerns
Title
#1-In the title appear Win and BreathWISE, both terms are not explained in the rest of the article. At least, Breath WISE should be explained as a structured approach to breathing techniques aimed at improving lung function, reducing stress and enhancing overall well-being. Contrariwise, both terms should be changed for “breathing”.

**Thank you for your comment. The BreathWISE was chosen as an acronym (see the capital letters in the title). Following your observation, to make it clearer, and to better summarize out finding, the following sentence has been added in the discussion:**

“*Although not directly aimed to compare different voluntary breathing techniques, our study suggests that the wisest breathing strategy during an incremental exercise would involve a gradual increase in the oral contribution to VE from the AT until the end of the effort.*”

#2- It is not well described whether the specific strategies lead to an improvement in exercise performance or whether they have been directed towards breathing therapies.

**Thank you, we agree. However, our study was just a preliminary mechanistic study. So that, our findings can act as hypothesis generating data for further experiments focused on different types of exercise training. This was also included in the discussion: *”These findings are in line with previous data recently published by Eser et al. 24 in a cohort of both healthy and heart failure patients. In their experiment, exercising the subjects at 50% of the maximal exercise in a 5-minutes constant workload testing, they observed very negligible differences in VE with eOR vs eNAS conditions (-0.5 L/min in the healthy subject’s group). These data, even in presence of slightly different experimental conditions (maximal incremental CPET vs submaximal workload test) suggests once again, the presence of more pronounced differences at higher exercise stages, as illustrated in the Figure 4 curves. This may suggest even greater differences in the presence of higher exercise intensities, as could occur in highly trained athletes, although this has not been investigated in the present study*.”**

#3- In the Abstract, the sentences “Twelve healthy subjects (28.6±5.2 y, 50% males) performed the 4 CPETs within one month. Variables were analyzed at rest, at anaerobic threshold (AT), at intermediate exercise steps, and at peak” that appear in Results, should be move to Methods in Abstract.

**Done as requested.**

#4- In the fifth line of the Results paragraph, in the Abstract, authors write “Moreover, peak inspiration and peak expiration time were augmented, while forced expiratory volume and vital capacity at rest were reduced. On opposite, only minor differences were detected at rest o AT”. If the variables forced expiratory volume and vital capacity were reduced at rest, what is the meaning of “On opposite, only minor differences were detected at rest or AT”? The authors should consider explaining or deleting this sentence.

**We have rephrased the sentence for clarity.**

Also the terms peak inspiration and peak expiratory time might be changed for “inspiratory and expiratory time to peak” for the sake of clarity.

**Thank you for your comment. However, we believe that the current formula is more appropriate since we do not want to indicate a “time to peak” delta, while the peak values of inspiratory and expiratory times. The same values were reported also in other manuscripts from our group (see for example PMID 33678608)**

#5- In Methods, the experimental subjects performed exercise in a cycle ergometer in which the subjects were allowed to see “rpm” (revolution per minute) but author did not tell if the rpm value was the same for the group or was different for each subject or was fixed during the four conditions. It ought be clarified.

**Thank you. The pedaling rhythm was maintained constant (60 to 65 rpm) in all the subjects/conditions. This is now better specified in the methods section.**

In statistical analysis the authors write that when the distribution of data belonged to a normal population they used mean±SD and when data belonged to a non-normal distribution they used median and interquartile. However normal and non-normal distributions were not assigned to the variables analysed in the article. It seems a stock phrase for statistic more than actual analysis.

**We acknowledge the reviewer's observation. The sentence in the Methods section remained by mistake, as all tested variables in our study were normally distributed. We have now corrected this in the revised manuscript.**

#6- RESULTS: The values of VO2 peak and VCO2 peak reveal a low training status of the subjects. Attending to VO2 and VCO2 peaks the tests should be classified as submaximal. Also HR peaks did not reach the maximum corresponding to the average age of the sample. The workload peak around 200 W is not a value of athletes. By other side, it is known that women show higher HR than men what may introduce a bias in the statiscal analysis of this variable.

**We agree that VO₂ and VCO₂ values alone are not outstanding, but they are in line for normal subjects. Moreover RQ (RER) is the more appropriate marker to evaluate maximality of the test. In our study, all subjects reached a maximal or near-maximal effort, as confirmed by RER >1.05 in all cases and by a maximal HR close to 100% of the predicted value, as stated in the manuscript (see also above). Regarding the population, we acknowledge that the subjects were not athletes; however, the protocol was specifically designed for a non-selected population rather than trained individuals.**

**Regarding differences in men and women, all the analyses were paired, so that they evaluated only intra patient differences. Moreover, we evaluated HR also as % of predicted, as also Reviewer 2 has suggested.**

#7 Figure 2. Both panels are lacking the units in the ordinate axis.

**Thank you for your observation. The values on Y axis refer to Borg scale, a 0-10 score to assess dyspnea and fatigue, with no specific unit.**

In FEV1 panel the ordinate values seem to be in percentage and FVC in litres; a Tiffenau index would be welcome. In the lower panel the ordinate scales should be in the same range to allow values to be compared. Provided the values of iT and eT (units?) in STD, pNAS and eOR conditions is difficult to believe in the significant differences among them, given the small sample and the standard deviation magnitudes. Further, in addition to the longer time for expiration as compared with inspiration, does not seem to there be significant differences in the eT values, including the eT peak value.

**Thank you for your contribution: we have corrected the figure by adding the missing units. The Tiffenau index was not calculated as the spirometry technique used was not a standard one, due to the use of the CPET mask instead of a mouthpiece to allow the protocol conditions. Therefore, the meaning of the index could, in our opinion, be misleading. Anyway, we have now added the value in the table according to your observation.**

Provided the ventilatory frequency (30.43 in eNAS vs 40 in eOR) and the PetCO2 peak (45 in eNAS vs 36.92 in eOR) of the eNAS condition (Table 2), it is not easy to explain how an increase in the end tidal CO2 is not correlated with higher ventilatory frequency in eNAS. It is well known than increase CO2 undergo an increase in ventilatory frequency. An eT longer than iT can help to explain the higher PetCO2 value in eNAS condition but not the change in ventilator frequency. May be these data deserve a better. explanation.

**You raised a very interesting point and I agree that a deeper discussion is needed. In reality, eNAS had a longer It (they have a longer distance and a greater air flow resistance between air and alveoli compared to all other conditions), so that the exercise performance is reduced as shown by peak workload and VO_2_. Moreover, the longer iT impedes to ventilation and its components (tidal volume and respiratory rate) to increase, so that PetCO_2_ must increase. Please note that this applies to peak exercise but also to exercise above AT. In brief, this condition (eNAS) is associated to a sort of respiratory limitation with ventilation unable to follow CO_2_ value and, consequently, to a lower exercise capacity.**

#8- Figure 4 The statistical differences applied to each workload step have no sense because increasing workload between steps (like Bruce Protocol) is mandatory. Moreover, this does not seem the main objective of the article but the different ventilation pattern among categories..

However, does there not be differences between each condition during the three first workload steps (in X axis). At least, differences between the eNAS and the other conditions in the last step of the CPET are clear. Again, the PetCO2 and the VE values deserve a better explanation.

**Thank you for your insightful comments. The figure does not compare different exercise steps; rather, our aim was to compare each subject’s performance at ISO-WATT moments. This approach was chosen to ensure that any observed differences in ventilation/VO_2_/VCO_2_ were not merely due to variations in exercise duration across experimental conditions, which would inherently be linked to different workload levels. Evaluating exercise time instead of wattage would have introduced this potential bias. This approach is the same used in a previous publication (**[**https://doi.org/10.1183/13993003.04473-2020**](https://doi.org/10.1183/13993003.04473-2020)**). We have revised the text to clarify this point accordingly. Specifically, we now report: “for eNAS, pNAS, and eOR tests, intermediate steps data were reported at the workload (Watt) corresponding to 25, 50 and 75% of maximal workload of the STD test, therefore the four conditions were compared iso-watt in each subject, as previously described^20^.”**

**See above for the PetCO_2_ differences.**

**Thank you again for your valuable feedback.**
