# Supplementary material for: Nasal vs. oral BREATHing WIn Strategies in healthy individuals during cardiorespiratory Exercise testing (BreathWISE)
Source: PLoS One. 2025 Jul 16;20(7):e0326661. doi: 10.1371/journal.pone.0326661 (PMC12266398; doi:10.1371/journal.pone.0326661)
Supplement: Attachment — Submitted filename: Analysis for the editor.docx [file pone.0326661.s003.docx]

Table 1 sphericity

|  | **Lambda di Wilks** | **ProbF** | **Mauchly** | **ProbChiSq** | **FValue** | **NumDF** | **DenDF** | **ProbF** | **Epsilon Greenhouse-Geisser** | **ProbFGG** | **Eta (η²p)** |
| --- | --- | --- | --- | --- | --- | --- | --- | --- | --- | --- | --- |
| ***FEV1 (L)*** | 0.4006 | **0.035** | 0.0041 | <.0001 | 15.58 | 3 | 33 | <.0001 | 0.3617 | 0.0017 | 0.5861 |
| ***FEV1%*** | 0.3361 | **0.016** | 0.0066 | <.0001 | 18.57 | 3 | 33 | <.0001 | 0.3672 | 0.0008 | 0.6280 |
| ***FVC (L)*** | 0.1504 | **0.001** | 0.1124 | 0.0007 | 23.30 | 3 | 33 | <.0001 | 0.4524 | <.0001 | 0.6793 |
| ***FEV1/FVC (%)*** | 0.5597 | **0.139** | 0.0038 | <.0001 | 6.93 | 3 | 33 | 0.0010 | 0.3606 | 0.0204 | 0.3866 |
| ***SBP rest (mmHg)*** | 0.3110 | **0.012** | 0.5862 | 0.3929 | 4.26 | 3 | 33 | 0.0119 | 0.7275 | 0.0234 | 0.2793 |
| ***DBP rest (mmHg)*** | 0.8397 | **0.647** | 0.3670 | 0.0827 | 0.35 | 3 | 33 | 0.7892 | 0.6146 | 0.6917 | 0.0309 |
| ***HR rest (bpm)*** | 0.9136 | **0.836** | 0.5072 | 0.2522 | 0.53 | 3 | 33 | 0.6626 | 0.7419 | 0.6120 | 0.0462 |
| ***VO_2_ rest (mL/min)*** | 0.8765 | **0.741** | 0.6639 | 0.5520 | 0.34 | 3 | 33 | 0.7942 | 0.8042 | 0.7513 | 0.0303 |
| ***VCO_2_ rest (mL/min)*** | 0.9608 | **0.945** | 0.8277 | 0.8710 | 0.17 | 3 | 33 | 0.9189 | 0.8942 | 0.9017 | 0.0148 |
| ***VE rest (L/min)*** | 0.8364 | **0.639** | 0.8150 | 0.8506 | 0.71 | 3 | 33 | 0.5511 | 0.9000 | 0.5380 | 0.0609 |
| ***RR rest*** | 0.8997 | **0.801** | 0.8102 | 0.8426 | 0.52 | 3 | 33 | 0.6740 | 0.8865 | 0.6530 | 0.0448 |
| ***TV rest (L)*** | 0.6723 | **0.289** | 0.2656 | 0.0244 | 0.89 | 3 | 33 | 0.4583 | 0.6908 | 0.4293 | 0.0746 |
| ***SpO_2_ rest (%)*** | 0.7188 | **0.373** | 0.6969 | 0.6218 | 1.06 | 3 | 33 | 0.3803 | 0.8157 | 0.3728 | 0.0877 |
| ***PetCO_2_ rest (mmHg)*** | 0.5965 | **0.180** | 0.7249 | 0.6803 | 1.87 | 3 | 33 | 0.1536 | 0.8289 | 0.1657 | 0.1454 |
| ***PetO_2_ rest (mmHg)*** | 0.6976 | **0.333** | 0.6919 | 0.6112 | 2.09 | 3 | 33 | 0.1210 | 0.8515 | 0.1325 | 0.1594 |
| ***VO_2_AT (mL/min)*** | 0.8760 | **0.772** | 0.4839 | 0.2752 | 0.27 | 3 | 30 | 0.8452 | 0.6858 | 0.7707 | 0.0265 |
| ***VO_2_AT (mL/Kg/min)*** | 0.8270 | **0.658** | 0.4595 | 0.2373 | 0.43 | 3 | 30 | 0.7327 | 0.6983 | 0.6649 | 0.0413 |
| ***VO_2_AT%*** | 0.8535 | **0.719** | 0.4815 | 0.2715 | 0.47 | 3 | 30 | 0.7074 | 0.7002 | 0.6424 | 0.0446 |
| ***HR AT (bpm)*** | 0.9527 | **0.938** | 0.5174 | 0.3315 | 0.17 | 3 | 30 | 0.9161 | 0.6903 | 0.8521 | 0.0167 |
| ***Workload AT (W)*** | 0.8944 | **0.814** | 0.3926 | 0.1480 | 0.33 | 3 | 30 | 0.8033 | 0.6211 | 0.7078 | 0.0320 |
| ***VCO_2_ AT (mL/min)*** | 0.5616 | **0.181** | 0.1763 | 0.0098 | 1.21 | 3 | 30 | 0.3226 | 0.5437 | 0.3143 | 0.1080 |
| ***VE AT (L/min)*** | 0.3941 | **0.049** | 0.2982 | 0.0610 | 1.97 | 3 | 30 | 0.1402 | 0.6184 | 0.1700 | 0.1644 |
| ***PetCO_2_ AT (mmHg)*** | 0.3597 | **0.035** | 0.6607 | 0.6060 | 6.77 | 3 | 30 | 0.0013 | 0.8041 | 0.0031 | 0.4038 |
| ***PetO_2_ AT (mmHg)*** | 0.3557 | **0.033** | 0.5772 | 0.4417 | 6.24 | 3 | 30 | 0.0020 | 0.7411 | 0.0058 | 0.3844 |

Delta

| ***Absolute differences*** | ***Test T 1 vs 0*** | ***Test T 2 vs 0*** | ***Test T 3 vs 0*** | ***Test T 2 vs 1*** | ***Test T 3 vs 1*** | ***Test T 3 vs 2*** |
| --- | --- | --- | --- | --- | --- | --- |
| ***FEV1 (L)*** | -0.1 (-0.25 ; 0.04) | -0.01 (-0.08 ; 0.05) | -1.34 (-2.08 ; -0.61) | 0.09 (-0.07 ; 0.25) | -1.24 (-1.94 ; -0.54) | -1.33 (-2.05 ; -0.61) |
| ***FEV1%*** | -2.08 (-5.42 ; 1.25) | -0.08 (-1.87 ; 1.7) | -32.58 (-48.88 ; -16.29) | 2 (-1.89 ; 5.89) | -30.5 (-46.44 ; -14.56) | -32.5 (-48.26 ; -16.74) |
| ***FVC (L)*** | -0.07 (-0.17 ; 0.04) | -0.04 (-0.12 ; 0.05) | -0.59 (-0.82 ; -0.37) | 0.03 (-0.1 ; 0.16) | -0.53 (-0.8 ; -0.25) | -0.56 (-0.74 ; -0.37) |
| ***FEV1/FVC (%)*** | -0.73 (-3.65 ; 2.18) | 0.39 (-0.98 ; 1.75) | -18.06 (-33.19 ; -2.93) | 1.12 (-2.29 ; 4.53) | -17.32 (-31.49 ; -3.16) | -18.45 (-33.45 ; -3.44) |
| ***SBP rest (mmHg)*** | -4.58 (-10.4 ; 1.24) | -11.25 (-16.52 ; -5.98) | -6.67 (-14.83 ; 1.5) | -6.67 (-11.61 ; -1.72) | -2.08 (-10.81 ; 6.64) | 4.58 (-3.71 ; 12.87) |
| ***DBP rest (mmHg)*** | 0.83 (-3.41 ; 5.08) | -0.83 (-5.87 ; 4.2) | 1.67 (-3.64 ; 6.97) | -1.67 (-5.33 ; 2) | 0.83 (-6.57 ; 8.23) | 2.5 (-4.73 ; 9.73) |
| ***HR rest (bpm)*** | -3.5 (-12.04 ; 5.04) | -3.33 (-13.23 ; 6.56) | -5.25 (-17.57 ; 7.07) | 0.17 (-5.28 ; 5.61) | -1.75 (-10.94 ; 7.44) | -1.92 (-11.27 ; 7.43) |
| ***VO_2_ rest (mL/min)*** | -23.5 (-104 ; 57) | 12.92 (-46.2 ; 72.03) | 3.5 (-97.41 ; 104.41) | 36.42 (-30.23 ; 103.06) | 27 (-63.92 ; 117.92) | -9.42 (-97.21 ; 78.37) |
| ***VCO_2_ rest (mL/min)*** | 1.33 (-67.68 ; 70.34) | 5.42 (-61.29 ; 72.12) | -15.75 (-99.36 ; 67.86) | 4.08 (-52.31 ; 60.48) | -17.08 (-95.17 ; 61.01) | -21.17 (-90.76 ; 48.43) |
| ***VE rest (L/min)*** | -0.46 (-2.75 ; 1.83) | 0.22 (-2.08 ; 2.51) | -1.18 (-3.79 ; 1.44) | 0.68 (-1 ; 2.35) | -0.72 (-3.04 ; 1.61) | -1.39 (-3.69 ; 0.91) |
| ***RR rest*** | 0.39 (-1.8 ; 2.58) | 0.89 (-2.25 ; 4.04) | -0.58 (-3.18 ; 2.01) | 0.5 (-2.09 ; 3.09) | -0.98 (-3.52 ; 1.57) | -1.48 (-4.51 ; 1.56) |
| ***TV rest (L)*** | -0.09 (-0.2 ; 0.02) | -0.05 (-0.22 ; 0.11) | 0.01 (-0.15 ; 0.17) | 0.03 (-0.05 ; 0.12) | 0.1 (-0.08 ; 0.28) | 0.06 (-0.12 ; 0.24) |
| ***SpO_2_ rest (%)*** | 0.17 (-0.49 ; 0.82) | -0.17 (-0.7 ; 0.36) | 0.33 (-0.35 ; 1.02) | -0.33 (-0.83 ; 0.16) | 0.17 (-0.64 ; 0.97) | 0.5 (-0.19 ; 1.19) |
| ***PetCO_2_ rest (mmHg)*** | -0.92 (-2.74 ; 0.91) | -0.58 (-2.86 ; 1.69) | 0.92 (-0.89 ; 2.72) | 0.33 (-1.18 ; 1.85) | 1.83 (0.35 ; 3.31) | 1.5 (-0.44 ; 3.44) |
| ***PetO_2_ rest (mmHg)*** | 0.25 (-3.15 ; 3.65) | 0.5 (-2.49 ; 3.49) | -2.67 (-6.16 ; 0.83) | 0.25 (-1.99 ; 2.49) | -2.92 (-6.1 ; 0.27) | -3.17 (-6.72 ; 0.38) |
| ***VO_2_AT (mL/min)*** | -8.58 (-204.3 ; 187.13) | 40.92 (-118.23 ; 200.06) | -36.91 (-312.51 ; 238.69) | 49.5 (-135.26 ; 234.26) | -48.27 (-200.44 ; 103.89) | -85 (-315.13 ; 145.13) |
| ***VO_2_AT (mL/Kg/min)*** | -0.18 (-2.84 ; 2.48) | 0.6 (-1.8 ; 3) | -0.81 (-4.64 ; 3.03) | 0.78 (-1.84 ; 3.41) | -0.86 (-2.88 ; 1.15) | -1.51 (-4.74 ; 1.72) |
| ***VO_2_AT%*** | -0.01 (-0.08 ; 0.06) | 0.02 (-0.04 ; 0.08) | -0.02 (-0.11 ; 0.08) | 0.03 (-0.04 ; 0.09) | -0.02 (-0.07 ; 0.04) | -0.04 (-0.12 ; 0.04) |
| ***HR AT (bpm)*** | -2.25 (-18 ; 13.5) | -2.17 (-12.3 ; 7.96) | -3.82 (-18.11 ; 10.48) | 0.08 (-11.04 ; 11.21) | -1.55 (-12.75 ; 9.66) | -3 (-13.08 ; 7.08) |
| ***Workload AT (W)*** | -3.75 (-21.75 ; 14.25) | 3.17 (-9.59 ; 15.92) | -2.27 (-26.87 ; 22.32) | 6.92 (-10.11 ; 23.94) | 0.45 (-12.69 ; 13.6) | -7.09 (-28.16 ; 13.98) |
| ***VCO_2_ AT (mL/min)*** | 52.33 (-328.92 ; 433.59) | 143.83 (3.59 ; 284.07) | -116.36 (-447.32 ; 214.59) | 91.5 (-307.23 ; 490.23) | -201.64 (-479.1 ; 75.82) | -284.18 (-673.67 ; 105.3) |
| ***VE AT (L/min)*** | -1.77 (-9.56 ; 6.03) | 0.85 (-4.98 ; 6.68) | -6.4 (-16.18 ; 3.38) | 2.62 (-4.05 ; 9.28) | -5.12 (-9.5 ; -0.73) | -7.44 (-14.18 ; -0.69) |
| ***PetCO_2_ AT (mmHg)*** | -0.58 (-2.29 ; 1.12) | -1.25 (-2.94 ; 0.44) | 2.55 (0.14 ; 4.96) | -0.67 (-2.11 ; 0.78) | 3.18 (1.46 ; 4.9) | 3.73 (1.36 ; 6.09) |
| ***PetO_2_ AT (mmHg)*** | -0.25 (-3.78 ; 3.28) | 1.33 (-1.2 ; 3.87) | -4.55 (-8.81 ; -0.28) | 1.58 (-0.82 ; 3.98) | -4.64 (-7.57 ; -1.7) | -6.18 (-9.66 ; -2.71) |

Delta %

| ***Percentage differences*** | ***Test T 1 vs 0*** | ***Test T 2 vs 0*** | ***Test T 3 vs 0*** | ***Test T 2 vs 1*** | ***Test T 3 vs 1*** | ***Test T 3 vs 2*** |
| --- | --- | --- | --- | --- | --- | --- |
| ***FEV1 (L)*** | -2.29 (-5.7 ; 1.13) | -0.08 (-1.88 ; 1.72) | -31.35 (-45.81 ; -16.88) | 2.57 (-1.73 ; 6.86) | -29.72 (-44.33 ; -15.11) | -31.4 (-45.56 ; -17.24) |
| ***FEV1%*** | -2.07 (-5.49 ; 1.34) | 0.11 (-1.75 ; 1.98) | -31.16 (-45.72 ; -16.61) | 2.54 (-1.75 ; 6.83) | -29.73 (-44.32 ; -15.14) | -31.41 (-45.54 ; -17.28) |
| ***FVC (L)*** | -1.49 (-3.9 ; 0.92) | -0.47 (-2.57 ; 1.63) | -13.37 (-17.96 ; -8.79) | 1.17 (-2.06 ; 4.4) | -11.91 (-17.26 ; -6.55) | -12.95 (-17.29 ; -8.62) |
| ***FEV1/FVC (%)*** | -0.76 (-4.01 ; 2.48) | 0.43 (-1.15 ; 2.02) | -20.47 (-37.55 ; -3.39) | 1.48 (-2.52 ; 5.47) | -20.15 (-36.59 ; -3.71) | -20.87 (-37.72 ; -4.02) |
| ***SBP rest (mmHg)*** | -3.31 (-8.16 ; 1.53) | -9.37 (-13.3 ; -5.44) | -4.38 (-11.33 ; 2.56) | -5.96 (-10.33 ; -1.59) | -0.67 (-8.62 ; 7.27) | 5.8 (-2.1 ; 13.69) |
| ***DBP rest (mmHg)*** | 1.19 (-4.58 ; 6.96) | -1.19 (-8.52 ; 6.14) | 3.32 (-5.01 ; 11.66) | -2.23 (-7.84 ; 3.37) | 3.04 (-7.62 ; 13.71) | 5.83 (-5.24 ; 16.9) |
| ***HR rest (bpm)*** | -2.62 (-11.7 ; 6.45) | -2.04 (-13.66 ; 9.57) | -3.94 (-17.4 ; 9.53) | 0.58 (-6.4 ; 7.57) | -1.19 (-12.6 ; 10.22) | -0.86 (-13.89 ; 12.18) |
| ***VO_2_ rest (mL/min)*** | -1.09 (-25.59 ; 23.41) | 5.57 (-13.27 ; 24.41) | 3.69 (-34.46 ; 41.84) | 15.84 (-7.42 ; 39.11) | 11.58 (-20.06 ; 43.21) | -2.04 (-25.32 ; 21.24) |
| ***VCO_2_ rest (mL/min)*** | 7.18 (-20.05 ; 34.42) | 7.2 (-16.59 ; 30.99) | -2.45 (-37.29 ; 32.39) | 4.79 (-12.23 ; 21.81) | -3.79 (-27.69 ; 20.12) | -7.15 (-27.93 ; 13.63) |
| ***VE rest (L/min)*** | 1.07 (-18.76 ; 20.9) | 5.83 (-15.27 ; 26.94) | -8.05 (-32.45 ; 16.35) | 7.04 (-8.43 ; 22.51) | -5.77 (-24.74 ; 13.2) | -10.79 (-28.12 ; 6.54) |
| ***RR rest*** | 7.61 (-6.51 ; 21.73) | 12.65 (-12.73 ; 38.04) | -3.69 (-17.99 ; 10.61) | 3.77 (-11.9 ; 19.43) | -8.21 (-24.55 ; 8.12) | -7.67 (-26.37 ; 11.04) |
| ***TV rest (L)*** | -7.34 (-22.44 ; 7.76) | -0.42 (-21.79 ; 20.94) | -0.23 (-23.25 ; 22.8) | 6.99 (-4.64 ; 18.63) | 12.44 (-12.86 ; 37.74) | 5.69 (-16.24 ; 27.61) |
| ***SpO_2_ rest (%)*** | 0.18 (-0.5 ; 0.85) | -0.16 (-0.71 ; 0.38) | 0.35 (-0.35 ; 1.05) | -0.34 (-0.84 ; 0.17) | 0.18 (-0.64 ; 1) | 0.52 (-0.19 ; 1.22) |
| ***PetCO_2_ rest (mmHg)*** | -2.29 (-7.96 ; 3.39) | -1.06 (-8.17 ; 6.04) | 3.31 (-2.83 ; 9.44) | 1.29 (-3.37 ; 5.95) | 5.95 (1.29 ; 10.62) | 5.04 (-1 ; 11.08) |
| ***PetO_2_ rest (mmHg)*** | 0.43 (-2.73 ; 3.59) | 0.66 (-2.15 ; 3.47) | -2.3 (-5.38 ; 0.78) | 0.3 (-1.75 ; 2.35) | -2.62 (-5.47 ; 0.23) | -2.84 (-6.03 ; 0.35) |
| ***VO_2_AT (mL/min)*** | 0.67 (-11.49 ; 12.83) | 4.16 (-6.22 ; 14.55) | -1.7 (-18.02 ; 14.62) | 5.31 (-6.51 ; 17.13) | -4.06 (-13.73 ; 5.62) | -5.69 (-18.76 ; 7.39) |
| ***VO_2_AT (mL/Kg/min)*** | 0.79 (-11.29 ; 12.87) | 4.17 (-6.22 ; 14.56) | -1.81 (-17.98 ; 14.36) | 5.15 (-6.6 ; 16.9) | -4.29 (-13.73 ; 5.15) | -5.82 (-18.71 ; 7.07) |
| ***VO_2_AT%*** | 0.54 (-11.67 ; 12.75) | 4.79 (-5.69 ; 15.27) | -0.9 (-17.32 ; 15.52) | 6.08 (-5.81 ; 17.98) | -3.23 (-12.79 ; 6.34) | -5.6 (-18.41 ; 7.2) |
| ***HR AT (bpm)*** | 0.27 (-10.53 ; 11.07) | -0.22 (-7.9 ; 7.46) | -0.93 (-11.36 ; 9.51) | 1.02 (-7.28 ; 9.33) | -0.21 (-8.89 ; 8.48) | -1.46 (-9.36 ; 6.44) |
| ***Workload AT (W)*** | -2.99 (-16.58 ; 10.6) | 4.62 (-7.77 ; 17) | 1.46 (-18.44 ; 21.36) | 9.63 (-1.79 ; 21.05) | 3.3 (-7.21 ; 13.81) | -4.43 (-17.58 ; 8.71) |
| ***VCO_2_ AT (mL/min)*** | 11.84 (-27.28 ; 50.96) | 10.34 (-0.29 ; 20.98) | -6.36 (-25.57 ; 12.85) | 14.41 (-10.1 ; 38.91) | -10.51 (-26.11 ; 5.1) | -14.89 (-32.44 ; 2.67) |
| ***VE AT (L/min)*** | -1.27 (-18.85 ; 16.31) | 5.3 (-7.51 ; 18.11) | -11.58 (-30.08 ; 6.92) | 11.07 (-5.9 ; 28.04) | -11.75 (-22.94 ; -0.57) | -16.77 (-30.27 ; -3.27) |
| ***PetCO_2_ AT (mmHg)*** | -1.34 (-5.44 ; 2.76) | -2.82 (-6.86 ; 1.22) | 6.27 (0.14 ; 12.41) | -1.35 (-4.87 ; 2.17) | 7.89 (3.76 ; 12.01) | 9.4 (3.39 ; 15.41) |
| ***PetO_2_ AT (mmHg)*** | -0.03 (-3.58 ; 3.53) | 1.39 (-1.23 ; 4.02) | -4.15 (-8.04 ; -0.27) | 1.54 (-0.75 ; 3.84) | -4.42 (-7.2 ; -1.64) | -5.74 (-8.92 ; -2.56) |

Table2 sphericity

|  | **Lambda di Wilks** | **ProbF** | **Mauchly** | **ProbChiSq** | **FValue** | **NumDF** | **DenDF** | **ProbF** | **Epsilon Greenhouse-Geisser** | **ProbFGG** | **Eta (η²p)** |
| --- | --- | --- | --- | --- | --- | --- | --- | --- | --- | --- | --- |
| ***SBP peak (mmHg)*** | 0.9298 | **0.876** | 0.4263 | 0.1410 | 0.37 | 3 | 33 | 0.7743 | 0.6930 | 0.7019 | 0.0326 |
| ***DBP peak (mmHg)*** | 0.7211 | **0.377** | 0.4865 | 0.2203 | 1.30 | 3 | 33 | 0.2923 | 0.6816 | 0.2940 | 0.1054 |
| ***HR peak (bpm)*** | 0.5430 | **0.123** | 0.0646 | <.0001 | 6.59 | 3 | 33 | 0.0013 | 0.4231 | 0.0173 | 0.3746 |
| ***HR peak (% pred)*** | 0.5448 | **0.125** | 0.0628 | <.0001 | 6.64 | 3 | 33 | 0.0012 | 0.4216 | 0.0171 | 0.3764 |
| ***VO_2_ peak (mL/min)*** | 0.3144 | **0.021** | 0.2268 | 0.0239 | 13.39 | 3 | 30 | <.0001 | 0.5262 | 0.0007 | 0.5726 |
| ***VO_2_ peak (mL/Kg/min)*** | 0.2816 | **0.008** | 0.4103 | 0.1233 | 16.44 | 3 | 33 | <.0001 | 0.6292 | <.0001 | 0.5991 |
| ***VO_2_ peak%*** | 0.2605 | **0.005** | 0.5315 | 0.2923 | 16.80 | 3 | 33 | <.0001 | 0.6997 | <.0001 | 0.6043 |
| ***VE/VCO_2_slope*** | 0.3562 | **0.021** | 0.6845 | 0.5955 | 5.54 | 3 | 33 | 0.0034 | 0.7851 | 0.0073 | 0.3350 |
| ***VO_2_/work*** | 0.8054 | **0.562** | 0.3174 | 0.0483 | 0.63 | 3 | 33 | 0.6030 | 0.5902 | 0.5262 | 0.0539 |
| ***VCO_2_ peak (mL/min)*** | 0.2838 | **0.008** | 0.2989 | 0.0385 | 16.64 | 3 | 33 | <.0001 | 0.5724 | 0.0001 | 0.6020 |
| ***VE peak (L/min)*** | 0.1670 | **0.001** | 0.2743 | 0.0277 | 33.83 | 3 | 33 | <.0001 | 0.5382 | <.0001 | 0.7546 |
| ***RR peak*** | 0.0648 | **<.0001** | 0.1511 | 0.0025 | 22.05 | 3 | 33 | <.0001 | 0.5657 | <.0001 | 0.6671 |
| ***RER*** | 0.3766 | **0.027** | 0.7631 | 0.7569 | 9.23 | 3 | 33 | 0.0001 | 0.8502 | 0.0004 | 0.4563 |
| ***TV peak (L/min)*** | 0.3151 | **0.012** | 0.4618 | 0.1853 | 9.48 | 3 | 33 | 0.0001 | 0.7574 | 0.0006 | 0.4628 |
| ***SpO_2_ peak*** | 0.7962 | **0.540** | 0.6964 | 0.6208 | 0.62 | 3 | 33 | 0.6066 | 0.8401 | 0.5806 | 0.0534 |
| ***PetCO_2_ peak (mmHg)*** | 0.1098 | **0.000** | 0.2585 | 0.0220 | 29.06 | 3 | 33 | <.0001 | 0.5749 | <.0001 | 0.7254 |
| ***PetO_2_ peak (mmHg)*** | 0.2751 | **0.007** | 0.1741 | 0.0045 | 21.15 | 3 | 33 | <.0001 | 0.5122 | <.0001 | 0.6578 |
| ***BR peak*** | 0.7871 | **0.519** | 0.0343 | <.0001 | 0.28 | 3 | 33 | 0.8414 | 0.3939 | 0.6459 | 0.0246 |
| ***Workload peak (W)*** | 0.4054 | **0.036** | 0.0756 | 0.0001 | 6.79 | 3 | 33 | 0.0011 | 0.4740 | 0.0125 | 0.3817 |
| ***Y intercept (L/min)*** | 0.6978 | **0.508** | 0.6257 | 0.6765 | 1.05 | 3 | 24 | 0.3880 | 0.7759 | 0.3787 | 0.1162 |

Delta

| ***Absolute differences*** | ***Test T 1 vs 0*** | ***Test T 2 vs 0*** | ***Test T 3 vs 0*** | ***Test T 2 vs 1*** | ***Test T 3 vs 1*** | ***Test T 3 vs 2*** |
| --- | --- | --- | --- | --- | --- | --- |
| ***SBP peak (mmHg)*** | -3.33 (-16.97 ; 10.3) | -1.67 (-14.61 ; 11.28) | -7.5 (-29.19 ; 14.19) | 1.67 (-13.86 ; 17.19) | -4.17 (-23.03 ; 14.69) | -5.83 (-20.03 ; 8.36) |
| ***DBP peak (mmHg)*** | -4.17 (-14.11 ; 5.77) | 0.42 (-7.06 ; 7.89) | -4.58 (-12.18 ; 3.01) | 4.58 (-2.25 ; 11.42) | -0.42 (-5.57 ; 4.73) | -5 (-10.59 ; 0.59) |
| ***HR peak (bpm)*** | 0.33 (-1.81 ; 2.47) | -0.92 (-3.95 ; 2.12) | -9.92 (-18.22 ; -1.62) | -1.25 (-3.52 ; 1.02) | -10.25 (-18.09 ; -2.41) | -9 (-16.86 ; -1.14) |
| ***HR peak (% pred)*** | 0.17 (-0.98 ; 1.32) | -0.51 (-2.14 ; 1.13) | -5.42 (-9.92 ; -0.91) | -0.67 (-1.9 ; 0.55) | -5.58 (-9.85 ; -1.31) | -4.91 (-9.18 ; -0.64) |
| ***VO_2_ peak (mL/min)*** | -56.25 (-142.79 ; 30.29) | 0.58 (-75.74 ; 76.91) | -379.27 (-566.65 ; -191.9) | 56.83 (-40.92 ; 154.59) | -310.91 (-494.43 ; -127.39) | -367.73 (-584.7 ; -150.76) |
| ***VO_2_ peak (mL/Kg/min)*** | -0.86 (-2.15 ; 0.43) | -0.08 (-1.34 ; 1.19) | -5.35 (-7.58 ; -3.12) | 0.78 (-0.77 ; 2.34) | -4.49 (-6.8 ; -2.19) | -5.28 (-7.92 ; -2.63) |
| ***VO_2_ peak%*** | -0.02 (-0.05 ; 0.01) | 0 (-0.04 ; 0.03) | -0.14 (-0.19 ; -0.08) | 0.02 (-0.02 ; 0.06) | -0.11 (-0.17 ; -0.06) | -0.13 (-0.2 ; -0.07) |
| ***VE/VCO_2_slope*** | 1.82 (-0.6 ; 4.23) | 0.49 (-1.9 ; 2.88) | -1.9 (-3.76 ; -0.04) | -1.33 (-2.85 ; 0.2) | -3.72 (-5.67 ; -1.77) | -2.39 (-4.33 ; -0.45) |
| ***VO_2_/work*** | -0.2 (-0.56 ; 0.16) | -0.25 (-0.72 ; 0.23) | -0.33 (-1 ; 0.35) | -0.05 (-0.49 ; 0.39) | -0.13 (-0.89 ; 0.63) | -0.08 (-0.58 ; 0.42) |
| ***VCO_2_ peak (mL/min)*** | -87.42 (-245.24 ; 70.4) | -12.25 (-136.53 ; 112.03) | -632.17 (-900.2 ; -364.13) | 75.17 (-84.91 ; 235.25) | -544.75 (-836.12 ; -253.38) | -619.92 (-929.52 ; -310.32) |
| ***VE peak (L/min)*** | -1.3 (-6.78 ; 4.18) | -3.39 (-8.25 ; 1.46) | -33.06 (-44.45 ; -21.67) | -2.09 (-7.39 ; 3.2) | -31.76 (-41.4 ; -22.12) | -29.67 (-40.86 ; -18.47) |
| ***RR peak*** | 1.05 (-1.24 ; 3.34) | -1.31 (-4.51 ; 1.89) | -10.89 (-16.14 ; -5.64) | -2.36 (-4.89 ; 0.17) | -11.94 (-15.29 ; -8.59) | -9.58 (-13.92 ; -5.25) |
| ***RER*** | -0.03 (-0.07 ; 0.02) | -0.02 (-0.06 ; 0.03) | -0.12 (-0.18 ; -0.06) | 0.01 (-0.04 ; 0.06) | -0.09 (-0.15 ; -0.03) | -0.1 (-0.16 ; -0.04) |
| ***TV peak (L/min)*** | -0.1 (-0.21 ; 0.01) | -0.01 (-0.09 ; 0.06) | -0.27 (-0.4 ; -0.14) | 0.09 (-0.05 ; 0.22) | -0.17 (-0.33 ; -0.02) | -0.26 (-0.41 ; -0.11) |
| ***SpO_2_ peak*** | 0.58 (-0.37 ; 1.54) | -0.08 (-1.45 ; 1.28) | 0.5 (-1.09 ; 2.09) | -0.67 (-1.86 ; 0.52) | -0.08 (-1.42 ; 1.26) | 0.58 (-0.94 ; 2.1) |
| ***PetCO_2_ peak (mmHg)*** | -1.33 (-2.92 ; 0.25) | -0.33 (-2.27 ; 1.61) | 7.75 (4.79 ; 10.71) | 1 (-0.41 ; 2.41) | 9.08 (6.52 ; 11.65) | 8.08 (4.68 ; 11.48) |
| ***PetO_2_ peak (mmHg)*** | 0.75 (-1.68 ; 3.18) | 0 (-1.82 ; 1.82) | -10 (-14.73 ; -5.27) | -0.75 (-2.33 ; 0.83) | -10.75 (-15.14 ; -6.36) | -10 (-14.38 ; -5.62) |
| ***BR peak*** | -0.68 (-5.14 ; 3.79) | 2.18 (-1.16 ; 5.53) | -2.81 (-19.26 ; 13.64) | 2.86 (-2.21 ; 7.93) | -2.13 (-19.44 ; 15.17) | -4.99 (-21.11 ; 11.13) |
| ***Workload peak (W)*** | -3.33 (-11.09 ; 4.42) | 0.67 (-4.32 ; 5.65) | -22.17 (-37.26 ; -7.08) | 4 (-4.44 ; 12.44) | -18.83 (-34.99 ; -2.68) | -22.83 (-41.55 ; -4.12) |
| ***Y intercept (L/min)*** | 0.13 (-2.25 ; 2.5) | 1 (-0.44 ; 2.44) | 1.64 (-0.74 ; 4.03) | 0.87 (-1.45 ; 3.19) | 1.63 (-1.5 ; 4.77) | 0.56 (-1.49 ; 2.6) |

Delta %

| ***Percentage differences*** | ***Test T 1 vs 0*** | ***Test T 2 vs 0*** | ***Test T 3 vs 0*** | ***Test T 2 vs 1*** | ***Test T 3 vs 1*** | ***Test T 3 vs 2*** |
| --- | --- | --- | --- | --- | --- | --- |
| ***SBP peak (mmHg)*** | -1.69 (-9.92 ; 6.55) | 0.45 (-8.11 ; 9) | -2.32 (-14.54 ; 9.9) | 3.42 (-7 ; 13.84) | 0.02 (-12.06 ; 12.09) | -3.05 (-10.37 ; 4.26) |
| ***DBP peak (mmHg)*** | -3.62 (-14.98 ; 7.75) | 1.56 (-6.9 ; 10.03) | -4.29 (-12.33 ; 3.76) | 7.29 (-2.56 ; 17.14) | 0.61 (-5.98 ; 7.21) | -5.38 (-11.44 ; 0.68) |
| ***HR peak (bpm)*** | 0.2 (-0.96 ; 1.36) | -0.49 (-2.1 ; 1.12) | -5.25 (-9.59 ; -0.91) | -0.69 (-1.89 ; 0.52) | -5.45 (-9.51 ; -1.39) | -4.77 (-8.91 ; -0.64) |
| ***HR peak (% pred)*** | 0.2 (-0.96 ; 1.36) | -0.49 (-2.1 ; 1.12) | -5.25 (-9.59 ; -0.91) | -0.69 (-1.89 ; 0.52) | -5.45 (-9.51 ; -1.39) | -4.77 (-8.91 ; -0.64) |
| ***VO_2_ peak (mL/min)*** | -2.71 (-6.72 ; 1.31) | -0.44 (-4.62 ; 3.74) | -16.83 (-23 ; -10.66) | 2.63 (-2.52 ; 7.79) | -13.82 (-20.8 ; -6.84) | -15.69 (-23.45 ; -7.94) |
| ***VO_2_ peak (mL/Kg/min)*** | -2.55 (-6.63 ; 1.53) | -0.44 (-4.63 ; 3.76) | -15.95 (-21.85 ; -10.06) | 2.49 (-2.77 ; 7.74) | -13.5 (-19.92 ; -7.09) | -15.23 (-22.32 ; -8.13) |
| ***VO_2_ peak%*** | -2.37 (-6.17 ; 1.44) | -0.26 (-4.45 ; 3.94) | -15.5 (-21.65 ; -9.35) | 2.44 (-2.69 ; 7.57) | -13.24 (-19.78 ; -6.7) | -14.94 (-22.17 ; -7.7) |
| ***VE/VCO_2_slope*** | 7.92 (-1.24 ; 17.09) | 2.76 (-6.67 ; 12.2) | -6.39 (-12.84 ; 0.07) | -4.5 (-9.71 ; 0.71) | -12.49 (-18.35 ; -6.64) | -8.01 (-14.74 ; -1.28) |
| ***VO_2_/work*** | -1.77 (-5.38 ; 1.84) | -2.29 (-7.14 ; 2.56) | -3.19 (-10.4 ; 4.02) | -0.4 (-5.09 ; 4.28) | -1.15 (-9.24 ; 6.95) | -0.94 (-6.43 ; 4.56) |
| ***VCO_2_ peak (mL/min)*** | -4.04 (-9.95 ; 1.87) | -1.3 (-6.61 ; 4.01) | -22.76 (-31.15 ; -14.37) | 3.6 (-3.89 ; 11.09) | -19.17 (-27.73 ; -10.6) | -21.39 (-30.32 ; -12.45) |
| ***VE peak (L/min)*** | -1.55 (-7.77 ; 4.68) | -3.89 (-9.74 ; 1.96) | -36.63 (-48 ; -25.25) | -1.8 (-8.69 ; 5.09) | -36.17 (-45.82 ; -26.52) | -33.91 (-45.38 ; -22.45) |
| ***RR peak*** | 2.98 (-3.31 ; 9.27) | -2.62 (-10.33 ; 5.09) | -26.84 (-40.38 ; -13.3) | -5.25 (-11.36 ; 0.86) | -29.88 (-39.54 ; -20.23) | -24.96 (-36.98 ; -12.95) |
| ***RER*** | -1.95 (-5.39 ; 1.5) | -1.2 (-4.97 ; 2.56) | -9.59 (-14.68 ; -4.5) | 0.94 (-3.46 ; 5.34) | -7.69 (-12.73 ; -2.64) | -8.37 (-13.36 ; -3.38) |
| ***TV peak (L/min)*** | -4.23 (-8.28 ; -0.18) | -0.95 (-4.69 ; 2.8) | -12.7 (-19.02 ; -6.38) | 3.78 (-1.6 ; 9.15) | -8.52 (-15.89 ; -1.14) | -11.67 (-18.22 ; -5.13) |
| ***SpO_2_ peak*** | 0.62 (-0.38 ; 1.61) | -0.07 (-1.49 ; 1.36) | 0.54 (-1.11 ; 2.19) | -0.67 (-1.9 ; 0.56) | -0.07 (-1.45 ; 1.31) | 0.63 (-0.95 ; 2.2) |
| ***PetCO_2_ peak (mmHg)*** | -3.47 (-7.36 ; 0.43) | -0.4 (-5.35 ; 4.56) | 21.02 (13.36 ; 28.69) | 3.28 (-1.05 ; 7.6) | 25.42 (18.76 ; 32.08) | 21.99 (13.31 ; 30.67) |
| ***PetO_2_ peak (mmHg)*** | 0.7 (-1.44 ; 2.84) | 0.04 (-1.59 ; 1.67) | -8.74 (-12.99 ; -4.5) | -0.61 (-1.97 ; 0.75) | -9.36 (-13.31 ; -5.4) | -8.78 (-12.75 ; -4.81) |
| ***BR peak*** | 0.36 (-13.65 ; 14.36) | 5.7 (-2.39 ; 13.8) | -14.6 (-65.94 ; 36.73) | 9.97 (-8.88 ; 28.82) | -7.08 (-60.27 ; 46.12) | -17.6 (-64.13 ; 28.94) |
| ***Workload peak (W)*** | -2.09 (-6.05 ; 1.87) | -0.27 (-2.65 ; 2.1) | -9.8 (-15.87 ; -3.74) | 2.26 (-2.8 ; 7.32) | -7.66 (-14.03 ; -1.29) | -9.27 (-16.65 ; -1.88) |
| ***Y intercept (L/min)*** | 34.15 (-259.91 ; 328.22) | 16.86 (-252.66 ; 286.38) | 184.57 (-42.77 ; 411.91) | -3.78 (-71.21 ; 63.64) | 11.16 (-85.25 ; 107.56) | -44.29 (-193.69 ; 105.11) |
